# *Staphylococcus haemolyticus* strains target mitochondria and induce caspase-dependent apoptosis of macrophages

**DOI:** 10.1007/s10482-012-9756-5

**Published:** 2012-06-02

**Authors:** Sylwia Krzymińska, Ewa Szczuka, Adam Kaznowski

**Affiliations:** Department of Microbiology, Faculty of Biology, A. Mickiewicz University, ul. Umultowska 89, 61-614 Poznań, Poland

**Keywords:** *Staphylococcus haemolyticus*, Genetic diversity, Cytotoxicity, Apoptosis

## Abstract

The aim of this study was to investigate the interaction of *Staphylococcus haemolyticus* strains with a macrophage cell line. Infection with the strains resulted in macrophage injury. All strains exhibited cytotoxic effects towards J774 cells. Moreover, the bacteria triggered apoptosis of the cells. The lowest apoptotic index did not exceed 21 %, whereas the highest reached 70 % at 24 h and 85 % at 48 h after infection. Incubation with the bacteria caused loss of mitochondrial membrane potential (ΔΨm) in macrophages. The pro-apoptotic activity of the strains was blocked by a pan-caspase inhibitor z-VAD-fmk, indicating the involvement of caspases in the bacteria-mediated cell death. We observed that the induction of macrophage apoptosis could constitute an important mechanism of pathogenesis by which *S. haemolyticus* strains evade host immune defences and cause disease.

## Introduction

Among the coagulase-negative staphylococci (CoNS), *Staphylococcus haemolyticus* strains are the second most commonly isolated bacteria that play important roles in hospital-acquired opportunistic infections. Infection with these bacteria may play a significant role in patients with underlying disease, such as those with prosthetic devices, surgical patients, individuals undergoing dialysis, or patients with diabetes. CoNS cause septicemia, endocarditis, otitis, urinary tract illnesses and peritonitis (Falcone et al. 2007; Falcone et al. 2004; Ertem et al. 2010). Previous studies have documented outbreaks or persistence of *S*. *haemolyticus* strains that affected patients in intensive care units (Klingenberg et al. 2007; Mazzariol et al. 2012). An association between liver abscess and colonic cancer and infection with *S*. *haemolyticus* strains has been noted (Gamberini et al. 2006).

The mechanisms of *S*. *haemolyticus* pathogenesis are still poorly understood. The genomes of *S*. *haemolyticus* clinical isolates contain genes encoding putative virulence factors, which include hemolysins, adhesins, exonucleases and proteases (Takueschi et al. 2005). The pathogenesis may be also related to the production of an extracellular polysaccharide slime that permits the bacteria to adhere to polystyrene surfaces and to colonize catheters and prosthetic heart valves (Spare et al. 2003). However, there is still a lack of knowledge regarding the contribution of these factors to the pathogenicity of *S*. *haemolyticus*.

Cell-mediated killing represents the major defense mechanism against host nonspecific immunity (Sansonetti and Di Santo 2007). Macrophages contribute to the primary line of innate defence against bacterial pathogens by providing their removal and destruction at the level of the epithelial barrier. Therefore, many bacterial pathogens have developed specific strategies to subvert the effective antimicrobial immune response of the cells in order to avoid the innate immune defence of the host. The ability of bacterial pathogens to promote apoptosis of immune cells may be important for bacterial survival and escape from the host immune defence and is implicated in the mechanism of pathogenesis (Sansonetti and Di Santo 2007; Böhme and Rudel 2009). Cells undergoing apoptosis show a characteristic sequence of morphological and biochemical features including membrane blebbing, cellular shrinkage and condensation of chromatin and degradation of DNA by cleavage to internucleosomal-sized fragments.

In this study, we analyzed interactions of *S*. *haemolyticus* strains with macrophages to understand better the pathogenic mechanisms of the bacteria. Moreover, we determined the clonal structure of the strains to detect whether one predominant clone is responsible for infections of many patients or genetically unrelated strains caused diseases.

## Materials and methods

### Bacterial strains

Thirty *S. haemolyticus* strains were used in the study. All isolates were identified to the species level by using biochemical tests of the API Staph identification system (bioMérieux). The strains were isolated from: blood, urine, a postoperative wound and skin (Table 1). The isolates were maintained at −75 °C in tryptic soy broth (TSB, Difco) containing 50 % (vol/vol) glycerol. *Escherichia coli* K-12 C600 strain was used as the negative control.

**Table 1:** *Staphylococcus haemolyticus* strains used in the study

Source of origin (number of strains)	Isolates number
Blood (15)	MPU Sh1, 6, 7, 11, 13, 16, 17, 19, 20, 22, 23, 24, 25, 26, 30
Wound (4)	MPU Sh4, 9, 15, 28
Secretion (4)	MPU Sh2, 8, 18, 29
Medical devices (3)	MPU Sh10, 12, 21
Urine (2)	MPU Sh3, 5
Skin (2)	MPU Sh14, 27

### Clonal analysis by REP-PCR typing

Bacterial DNA was isolated with genomic DNA Plus kit (A&A Biotechnology, Poland). The REP-PCR method uses primers complementary to repetitive extragenic palindromic (REP) elements of bacterial genomic DNA (Versalovic et al. 1991). The amplicons were electrophoresed in 1.5 % agarose gels. The DNA in gels was stained with ethidium bromide, visualized on a UV light transilluminator and documented with the V.99 Bio-Print system (Vilber Lourmat, Torcy, France). Computer analysis was carried out by using GelCompar II (version 3.5; Applied Maths, Belgium) software. The similarity between fingerprints was calculated with the Dice coefficient. Cluster analysis was performed by using the unweighted pair-group method with average linkages (UPGMA).

### Macrophage cell line

The murine macrophage cell line J774 was maintained in growth medium (GM), containing RPMI 1640 supplemented with 10 % heat-inactivated fetal calf serum, gentamicin (5 μg/ml) and 2 mM l-glutamine. Cells were seeded in 100 μl of suspension (1 × 10^4^ cells per well) and incubated at 37 °C in the atmosphere of 5 % CO_2_ (Krzymińska et al. 2009).

### Cytotoxic activity

The bacteria were grown in TSB on a rotary shaker (150 rpm) at 37 °C for 24 h and centrifuged at 2,000×*g* for 20 min. The supernatants were sterilized through 0.22 μm-pore size membrane filters Millex-GV (Millipore). Monolayers of J774 cells were incubated with 100 μl of bacterial culture filtrate for 24 h. Some wells were incubated with GM and *E. coli* K-12 C600 as negative controls. Cytotoxicity was determined quantitatively based on MTT [3-(4,5-dimethylthiazol-2-yl)-2,5 diphenyltetrazolium bromide] (Sigma) uptake and reduction in tetrazolium-based colorimetric assay as described previously (Krzymińska et al. 2009).

Additionally, the number of viable cells was assessed by trypan blue exclusion. The suspension of trypsynized cells was stained with 0.1 % trypan blue (Sigma) for 3 min at room temperature and counted in a hemocytometer. The cells that excluded the stain were considered as viable, whereas those stained blue were considered dead.

### Cell-contact and extracellular hemolytic activity

To obtain a quantitative measure of hemolytic activity, bacterial cell suspension and extracellular supernatants were utilized. The cells from tryptic soy agar (TSA, Difco) were washed in PBS and diluted to optical density at 600 nm (OD_600_) = 0.3 (Krut et al. 2003). Fresh group 0 human blood was obtained from volunteer donors at a Blood Center. The plasma was discarded following centrifugation (250×*g* for 10 min) and erythrocytes were washed three times with sterile PBS. An equal volume of 1 % (vol/vol) erythrocyte solution was mixed with bacterial culture supernatant or suspension containing 1 × 10^7^ bacterial cells. After 1-h incubation at 37 °C, the mixture was centrifuged (250×*g* for 10 min) and further incubated for 3 h. The absorbance of supernatants was measured at 540 nm. Control tubes for spontaneous hemolysis contained 1 % erythrocyte solution and distilled water. The results were expressed as the percentage of total (100 %) hemolysis (Shimuta et al. 2009).

### Infection conditions

J774 cells (1 × 10^4^ cells per well) were infected with *S. haemolyticus* cell suspension diluted in GM to a concentration of 1 × 10^7^/ml. The viability of the bacteria was determined by plating dilutions on TSB agar and counting CFU/ml. Infection was performed at MOI (number of cells: bacteria ratio) of 1:100 for 90 min at 37 °C (Krut et al. 2003). Next, the monolayer was washed with PBS (Phosphate-buffered saline, Biomed) and incubated with GM containing 100 μg/ml gentamicin for 2 h at 37 °C. After washing three-times with PBS, the cells were incubated in the medium without gentamicin (Alexander et al. 2003).

### Determination of the percentage of apoptotic cells by fluorescence microscopy

The monolayer after infection was double stained with a solution containing 100 μg/ml of acridine orange (AO) and ethidium bromide (EB) for 2 min and visualized by Zeiss confocal laser-scanning microscopy. The characteristics of the cells were recorded according to the colour and structure of the chromatin and cells assigned to three different groups: viable, apoptotic and necrotic cells (Krzymińska et al. 2011). We determined apoptotic (AI) and necrotic indexes (NI) as the percentage of apoptotic and necrotic cells, respectively.

To determine the involvement of caspases in *S. haemolyticus*-induced apoptosis, J774 cells were incubated with a broad-spectrum caspase inhibitor Z-Val-Ala-Asp(*O*-methyl)-fluoromethylketone (z-VAD-fmk, R&D Systems) for 3 h prior to the infection and continued for 48 h after the infection. After 24 and 48 h the infected cells were examined by acridine orange and ethidium bromide staining.

### Assessment of DNA fragmentation

Degradation of nuclear DNA by cleavage to internucleosomal-sized fragments can be used as a biochemical marker of apoptosis. DNA was isolated from the infected and control cells 24 and 48 h after gentamicin treatment and fragmentation was assessed according to the method described previously (Krzymińska et al. 2011).

### Determination of mitochondrial transmembrane potential

Mitochondrial transmembrane potential (Δψ) was analysed using the Δψ-sensitive dye tetramethylrhodamine ethyl ester (TMRE, Sigma) as described previously (Krzyminska et al. 2011). In brief, infected cells were trypsinized and stained with 10 nM TMRE for 2 min at room temperature. After washing with PBS, the cells were imaged with an Axiovert 200 M (Zeiss) laser scanning confocal microscope at exitation λ 568 nm and emission λ > 590 nm by using a long-pass emission filter (Krzymińska et al. 2011).

### Statistical analysis

The values of the percentage cytotoxicity, viability, AI, NI and Δψ were determined in triplicate wells, and the data represent the means ± standard deviations (SD) from at least two separate experiments. A statistical analysis of significance was done by using a one-way analysis of variance (ANOVA) followed by Tukey’s HSD post hoc test for group comparison. The linear regression analysis was used to examine pairwise correlation between the Apoptotic Index, cytotoxicity and ΔΨ_m_, thus the Pearson correlation coefficient was determined. *P* values of < 0.05 were considered statistically significant.

## Results

### Clonal analysis

**Fig. 1:**
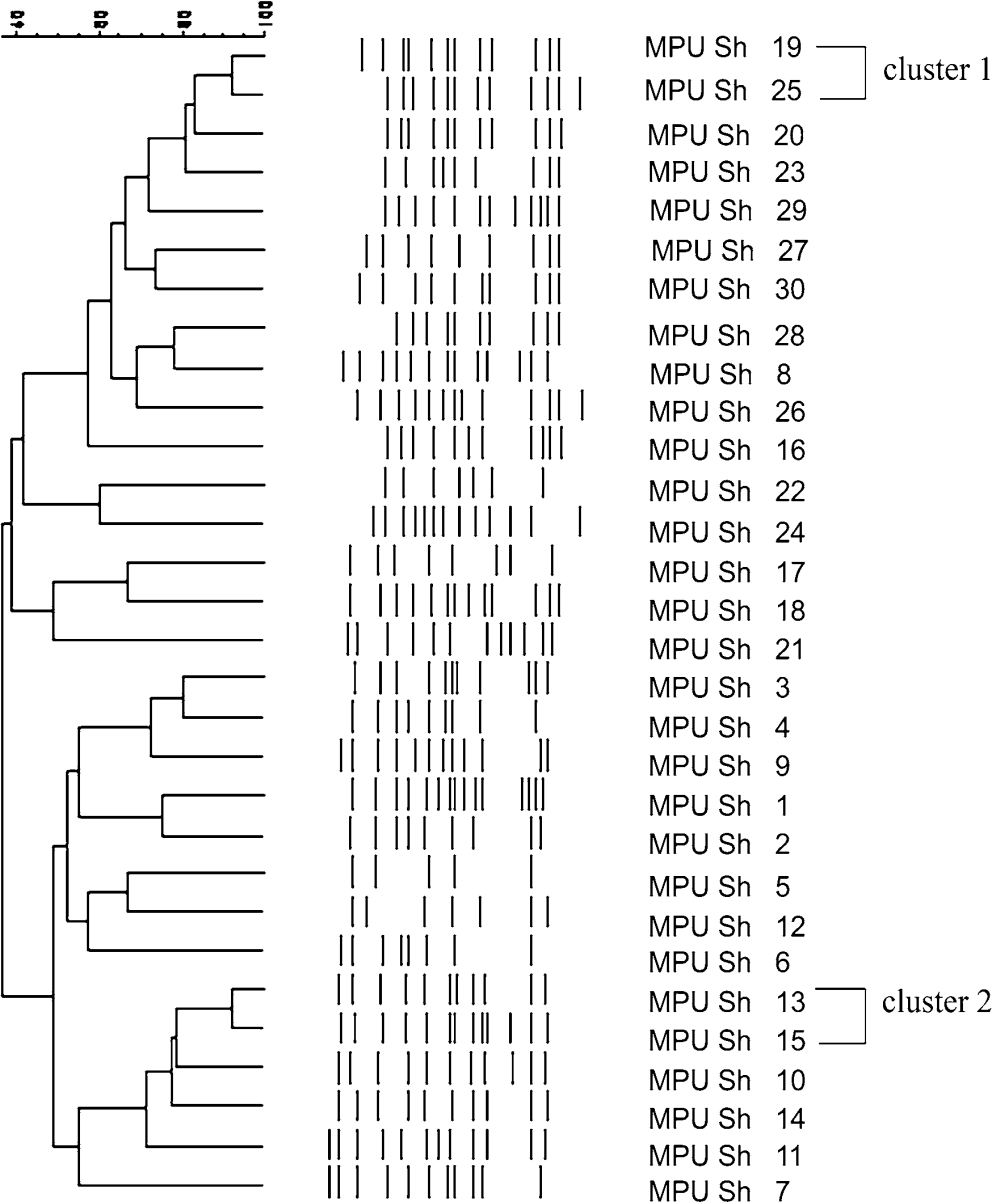
Dendrogram generated from REP-PCR fingerprint patterns of *Staphylococus haemolyticus* strains by the unweighted pair-group method (UPGMA) with arithmetic means. The Dice band-based similarity coefficient was calculated with band position tolerance of 1 %. The *scale bar* represents similarity

A dendrogram based on the REP-PCR analysis of DNA from the *S. haemolyticus* strains studied is presented in Fig. 1. We found various REP-PCR banding patterns from 7 to 15 bands (perpendicular lines). Only two clusters contained genetically closely related strains. The highest degree of similarity (*S* = 92 %) was obtained for two strains of *S. haemolyticus* isolated from the blood of two patients. Hospitalization of these patients took place at different times but at the same hospital ward. The second cluster (*S* = 91 %) was composed by two strains isolated from bedsore and blood. These strains were isolated from two patients treated in different hospital wards. The remaining strains showed distinct REP-PCR fingerprint patterns and were considered genetically unrelated. The fact that only two clusters were identified along with 26 unique genotypes indicates a large genetic diversity among *S. haemolyticus* isolates obtained from patients treated in a single hospital.

### Cytotoxic activity of *S. haemolyticus* strains

**Fig. 2:**
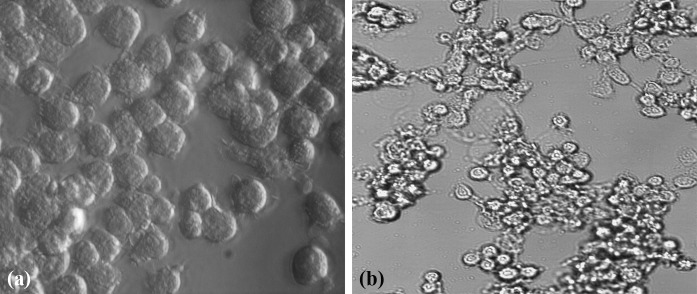
Cytotoxic effect of *S. haemolyticus* strains to J774 cells. The monolayer was incubated with culture medium (**a**), *S. haemolyticus* MPU Sh7 strain (**b**) and observed using an inverted microscope

The capacity of cell-free supernatants to impair J774 cell viability was determined at 24 h. The culture medium and the non-pathogenic *E. coli* K-12 C600 did not exhibit cytotoxic effects on J774 cells (Fig. 2a). All strains of *S. haemolyticus* exhibited cytotoxic effects which were evident by detachment of the cells from the surface of the wells (Fig. 2b). Three strains (10 %) showed the low cytotoxic activity (Table 2). By contrast, high activity was observed in culture supernatants of 7 (23 %) isolates originating from blood, wounds and respiratory secretions.

**Table 2:** The comparison of apoptotic index of J774 cells infected with *S. haemolyticus* strains at 24 and 48 h without and with the pan-caspase inhibitor, extracellular cytotoxic and hemolytic activity, and transmembrane mitochondrial potential (ΔΨ_m_)

Strain number	Apoptotic index^a^ (%)		Cytotoxic activity^b^ (%)	Hemolytic activity^c^ (%)	ΔΨ_m_^d^ × 10^2^ (F.U)
	24 (h)	48 (h)			
MPU Sh4	49.7/6.8	83.1/15.3	61.7	26.9	25.8/15.4
MPU Sh9	48.4/7.1	81.3/14.8	61.2	15.3	29.6/7.9
MPU Sh16	47.2/6.3	81.6/15.1	49.1	5.7	22.8/14.1
MPU Sh7	47.1/9.1	84.6/14.9	59.4	23.4	35.9/7.1
MPU Sh14	46.8/7.2	80.7/11.7	58.1	98.0	27.3/12.7
MPU Sh3	45.1/8.7	78.1/15.1	36.2	18.8	32.7/10.9
MPU Sh13	43.7/6.1	81.2/14.6	52.7	6.8	24.9/8.9
MPU Sh24	42.8/7.5	79.6/11.8	46.5	5.4	21.9/9.1
MPU Sh29	42.1/6.4	78.3/15.2	51.3	41.3	26.1/13.2
MPU Sh23	49.2/8.9	83.7/14.2	63.1	11.9	33.1/9.2
MPU Sh28	39.3/6.8	81.6/11.8	48.6	39.3	34.1/9.8
MPU Sh30	38.1/6.1	81.1/12.6	41.2	37.3	30.9/9.7
MPU Sh10	37.8/7.1	69.2/14.9	38.9	29.5	39.7/11.2
MPU Sh8	33.7/6.2	78.1/13.7	36.1	2.5	26.9/9.0
MPU Sh15	32.6/6.7	77.9/15.3	25.7	4.0	31.6/11.1
MPU Sh22	31.7/7.1	75.1/11.9	35.6	11.8	30.1/22.6
MPU Sh2	28.6/9.1	74.9/12.8	30.1	4.8	56.5/17.3
MPU Sh27	27.1/8.9	71.4/14.6	16.9	7.6	39.1/29.1
MPU Sh21	25.7/7.6	63.4/11.7	13.6	31.4	56.5/18.7
MPU Sh11	25.1/6.7	56.3/11.9	12.7	5.7	53.2/37.6
MPU Sh18	24.4/6.1	52.8/12.4	31.7	13.9	40.8/31.3
MPU Sh19	21.2/8.9.	63.9/15.2	21.7	11.2	38.9/21.6
MPU Sh20	20.8/6.4	48.8/11.8	19.5	6.8	41.7/26.4
MPU Sh1	19.5/6.7	65.4/15.1	10.3	45.5	41.8/10.2
MPU Sh26	18.9/8.4	66.8/14.9	18.4	4.9	53.1/19.1
MPU Sh12	18.1/6.2	41.7/11.8	17.1	6.1	44.6/29.8
MPU Sh17	17.8/6.3	37.2/12.3	8.7	3.5	55.8/28.6
MPU Sh25	16.4/6.5	62.1/15.1	27.1	8.4	43.7/16.1
MPU Sh5	16.1/6.1	43.2/11.9	11.4	3.6	52.7/32.6
MPU Sh6	15.6/7.3	39.1/11.7	15.3	27.5	54.1/25.7
*E. coli* K-12 C600	5.7/ND	11.9/ND	0	0	52.7/41.3

*ND* not determined

^a^The percentage of apoptotic cells after AO and EB staining at 24 and 48 h after infection without pan-caspase inhibitor/with the inhibitor

^b^The percentage of extracellular cytotoxicity measured by MTT assay

^c^The percentage of total extracellular hemolysis, compared to 100 % lysis in distilled water was performed at 4 h after infection by using a suspension of 1 % human erythrocytes

^d^TMRE fluorescence intensity per 25 cells at 24/48 h

The trypan blue exclusion assay revealed that the percentage of infected cells that remained viablee at 24 h ranged between 41 ± 2.1 and 87 ± 4.7 %. The viability decreased to values between 25 ± 3.7 and 65 ± 2.8 % at 48 h. The control J774 cells incubated with growth medium alone had viability of 94 ± 1.2 and 91 ± 1.8 % at 24 and 48 h, respectively.

### Hemolytic activity

The results showed that *S. haemolyticus* strains produced extracellular toxins which were able to lyse human erythrocytes (Table 2). The hemolytic activity ranged between 2.6 and 97.1 %. High activity was observed for one (3 %) strain. Low activity was demonstrated by 17 (57 %) strains. We observed a low percentage of cell-contact hemolysis, which was in the range from 1.7 to 7.2 %. The non-pathogenic *E. coli* strain K-12 C600 did not demonstrate hemolytic activity.

### Assessment of apoptosis and necrosis of infected J774 cells

**Fig. 3:**
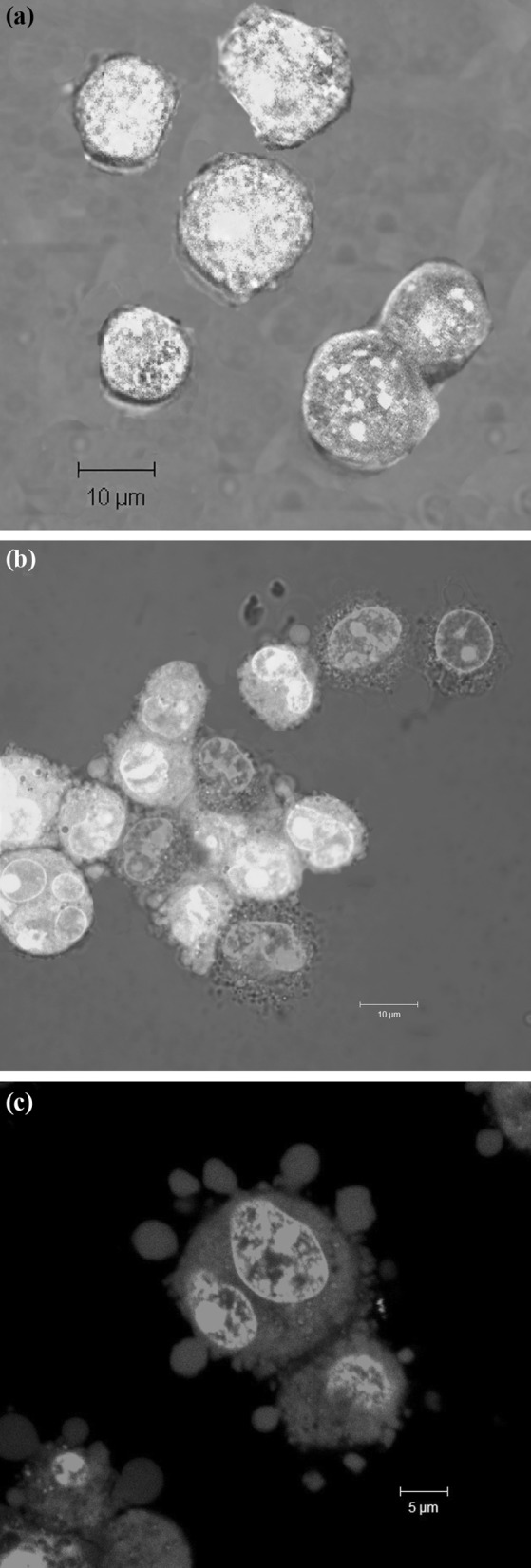
Apoptosis of J774 cells during *S. haemolyticus* infection. The macrophages were incubated with: culture medium (**a**), *S. haemolyticus* MPU Sh7 (**b**, **c**), cells with apoptotic bodies (**c**). The cells were observed using a laser scanning confocal microscope after AO and EB staining (*green cells*—live, *red*—apoptotic). (Color figure online)

Morphological evidence of apoptosis and necrosis of *S.* *haemolyticus*-infected cells was observed after AO and EB staining followed by confocal microscope analysis. Double staining revealed loss of cell membrane integrity and allowed discrimination between viable, apoptotic and necrotic cells. AO stains live cells and renders the nuclei of live cells green (Fig. 3a). EB is taken up by the cells only when cytoplasmic membrane integrity is lost, staining the nuclei of apoptotic cells red (Fig. 3b) with apoptotic bodies (Fig. 3c). The apoptotic indexes (AI) varied among the strains (Table 2). The highest AI, ranging from 41.2 ± 3.4 to 49.7 ± 4.1 % at 24 h after infection, was observed in phagocytes incubated with 10 (33 %) strains. In contrast, the lowest AI, between 15.6 ± 1.8 and 21.2 ± 2.1 %, was caused 9 (30 %) strains. The percentage of apoptotic cells increased at 48 h after the infection. The highest AI, ranging from 77.9 ± 4.1 to 84.6 ± 3.2 %, was observed in macrophages infected with 13 (43 %) strains whereas the lowest AI, ranging from 37.2 ± 2.7 to 48.8 ± 1.3 %, was revealed by J774 cells incubated with 5 (17 %) strains. The AI were reduced in the presence of the pan-caspase inhibitor to the range between 6.4 and 9.1 % at 24 h, and 11.7 and 15.3 % at 48 h, suggesting that apoptosis depends on caspase activation.

Twenty-two (73 %) and 24 (80 %) *S.* *haemolyticus* strains caused necrosis, respectively at 24 and 48 h. The highest NI, ranging from 9.1 ± 1.3 % to 17.6 ± 2.1 % at 24 h, was detected for 5 (17 %) strains. At 48 h the NI decreased for 9 (30 %) strains, and was between 11.7 ± 1.8 and 24.4 ± 3.1 %. These strains were isolated from urine (MPU Sh3, 5), blood (MPU Sh19, 20), drain (MPU Sh12) and skin (MPU Sh14, 27), a catheter (MPU Sh21) and respiratory secretion (MPU Sh18).

DNA fragmentation is a key feature of apoptosis. We observed fragmentation of nuclear DNA in J774 cells infected with 13 (43 %) strains at 24 h after infection (not shown). At 48 h, fragmentation was observed to be caused by 22 (73 %) strains.

### Mitochondrial transmembrane potential (ΔΨ_m_)

**Fig. 4:**
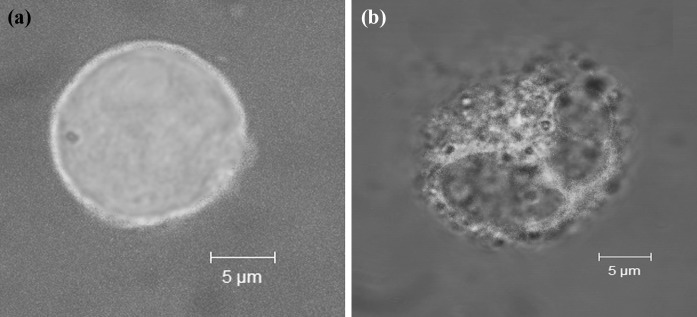
Mitochondrial membrane potential of J774 cells at the single cell level after 24 h-incubation with: culture medium (**a**), *S. haemolyticus* MPU Sh 7 (**b**). The infected cells were trypsinized and stained with 10 nM TMRE. The fluorescence was visualized by laser confocal microscopy

To gain insights into the pathway involved in *S. haemolyticus*-induced apoptosis of macrophages, we determined whether the process involved mitochondrial damage by the collapse of transmembrane potential. We therefore used a mitochondrion-selective fluorescent dye, whose uptake depends on an intact membrane. The dye exhibits changing colour intensity depending on Δψ. An analysis at the single cell level revealed a large difference of TMRE-fluorescence intensity between live and infected apoptotic J774 cells (Fig. 4a, b). The highest fluorescence intensity, ranging from 197 ± 15.7 to 210 ± 21.3 F.U, was observed in live cells. The intensity decreased to the range between 31.4 ± 11.3 and 36.1 ± 13.7 F.U in apoptotic and necrotic cells. The cationic dye accumulates in the mitochondria in proportion to Δψ and therefore the decrease in fluorescence intensity indicates depolarization of the mitochondrial membrane. We examined TMRE fluorescence in 25 cells in order to assess average ΔΨ_m_ in *S. haemolyticus*-infected cells (Table 2). High dissipation of fluorescence intensity was observed in cells infected with 7 (23 %) and 12 (40 %) strains at 24 and 48 h, respectively. The highest reduction of ΔΨ_m_ was visible as a lowering of fluorescence intensity to 890 ± 23.6 F.U on 25 cells. J774 cells incubated with *E. coli* K-12 C600, as a negative control, revealed 5.2 ± 0.7 × 10^3^ and 4.1 ± 0.3 × 10^3^ F.U, respectively at 24 and 48 h. The Pearson linear correlation test revealed positive correlations between AI of infected J774 cells and cytotoxic activity (*r* = 0.64, *p* < 0.01), and between AI and loss of ΔΨ_m_ (*r* = −0.57, *p* < 0.01) at 24 h.

## Discussion

The present study provides evidence that infection with *S. haemolyticus* strains caused alterations of macrophages and induced apoptosis of the cells. While *S. haemolyticus* is often considered to be of low virulence, there are also reports for its association with increased mortality (Worth and Slavin 2009). Phagocytes, such as macrophages, are essential effectors of the immune response against pathogenic bacteria. Therefore, the ability of pathogenic strains to circumvent the effector functions of the cells could be an important mechanism for the successful establishment of infection (Rudel et al. 2010). The results presented in the present study provide evidence that all *S. haemolyticus* strains were cytotoxic to macrophages. We observed that 23 % of them had cytotoxic activity which caused destruction above 50 % of macrophages. Previously, Zell et al. (2008) have analyzed the cytotoxic activity of CoNS isolated from food, other than *S. haemolyticus*. They reported that 18 of 35 strains were toxin positive and some of them produced more than one toxin. The most prevalent staphylococcal enterotoxins (SE) were SED and SEH, whilst two strains produced exfoliative toxin A. Vasconcelos et al. (2011) observed that 40 % of *S. haemolyticus* strains that originated from newborns hospitalized at a neonatal unit contained staphylococcal enterotoxins G (SEG) genes. High cytotoxic activity (more than 50 %) to keratinocytes has also been observed for 7 of 35 of *Staphylococcus aureus* clinical isolates (Krut et al. 2003). In the present study, we noticed positive correlations between cytotoxic and hemolytic activities for 9 (30 %) strains which suggests that cytotoxicity was evoked by hemolysins or pore-forming toxins. Previously, Butt et al. (1998) have reported that *S. haemolyticus* isolates from patients with chronic orofacial muscle pain produce δ hemolysins.

In the present study we observed in the trypan blue exclusion assay that infection with *S. haemolyticus* strains induced the cell death in macrophages in the range approximately from 13 to 59 and 35 to 75 % at 24 and 48 h, respectively. Fluorescent microscope observations after AO/EB staining revealed that the strains caused cell death by apoptosis. The apoptotic activity of *S. haemolyticus* strains varied among different isolates. High apoptotic activity, above 60 %, was observed for 10 (33 %) strains at 24 h and this increased to a range from 78 to 85 % for 13 (43 %) strains at 48 h. These strains originated from blood (6), wounds (4), secretions (2), skin and urine. This suggests this is a mechanism to subvert the functions of macrophages. The induction of cell death may suppress the effective antimicrobial immune responses of macrophages to avoid the innate immune defence of the host. The formation of apoptotic cells was prevented by preincubation with a pan-caspase inhibitor, indicating that caspases are involved with *S. haemolyticus*-induced cell death. Previously, Haslinger-Löffler et al. (2005) have observed a similar apoptotic activity to endothelial cells induced by clinical *S. aureus* isolates. The production of virulence factors by *S. haemolyticus* strains has not been clearly defined yet. The whole genome sequencing and comparative genomics of *Staphylococcus* spp. have identified genes of *S. haemolyticus* strains coding for some putative factors (Takueschi et al. 2005). However, there is still a lack of knowledge regarding the contribution of these factors to the pathogenesis of the bacteria (Otto 2004). In the present study, the Pearson linear correlation test revealed positive correlations between AI of infected J774 cells and cytotoxic activity, which suggested that apoptosis of the cells was associated with the cytotoxic activity of *S. haemolyticus* strains. Production by CoNS of extracellular δ-toxins and proteases such as metalloproteases and serine proteases which could exert cytotoxic activity has been previously observed (Harris and Richards 2006). In addition, strains of *S. haemolyticus* carry genes encoding capsular polysaccharide (CP). The *S. haemolyticus* CP has antiphagocytic properties and capsulated strains are resistant to opsonophagocytic killing by human neutrophils (Flauhaut et al. 2008).

There is increasing evidence that apoptosis can be triggered by a wide range of bacterial pathogens which have evolved different survival strategies, leading to the development of infection symptoms but there is little data concerning *Staphylococcus* spp. strains. Essmann et al. (2003) have reported that α-toxin, the major hemolysin of *S. aureus*, induces pore-forming cytotoxicity, which evokes apoptosis of breast carcinoma cells. These strains also secrete Panton-Valentine leukocidin (PVL), a pore-forming toxin which induces neutrophil cell death by apoptosis or necrosis, depending on PVL concentration (Genestier et al. 2005). Haslinger-Löffler et al. (2005) have suggested that *S. aureus* strains with cytotoxic and invasive activity induce caspase-dependent apoptosis of endothelial cells. This would explain the ability of the bacteria to invade the circulation from localized sites of infection and to disseminate systemically.

Mitochondrial dysfunction plays the main role in the regulation of apoptotic cell death (Rudel et al. 2010). The main step in mitochondrion-regulated apoptosis is the permeabilization of the outer membrane accompanied by loss of ΔΨ_m_, which is used as an indicator of apoptosis. We observed that macrophage cell death due to *S. haemolyticus* strains was provoked by perturbation of mitochondrial transmembrane potential. The potential significantly decreased to 22 and 9 F.U at 24 and 48 h after infection. It has been demonstrated that mitochondria are the main target of bacterial proteins that are transferred to host cells during infection (Rudel et al. 2010). Several toxins in a purified form associated with host cells target to mitochondria, inducing ΔΨ_m_ dissipation, cytochrome *c* release and apoptosis. These include α-toxin and PVL of *S. aureus* and pneumolysin of *Streptococcus pneumoniae* (Genestier et al. 2005; Rudel et al. 2010). All of these toxins create homo-multimers which form membrane pores.

In this study, we determined the clonal relatedness of *S. haemolyticus* strains isolated from human specimens. The majority of the strains had unique REP-PCR fingerprint patterns and this indicated that most of the patients were infected with clonally unrelated strains.

The results suggest that infection with *S. haemolyticus* strains induce apoptosis of host macrophages. The ability of the strains to circumvent the effector function of the phagocytic cells may be an important virulence mechanism of pathogenesis for the persistance and dissemination of the bacteria in the host.
